# Application of deep learning on mammographies to discriminate between low and high-risk DCIS for patient participation in active surveillance trials

**DOI:** 10.1186/s40644-024-00691-x

**Published:** 2024-04-05

**Authors:** Sena Alaeikhanehshir, Madelon M. Voets, Frederieke H. van Duijnhoven, Esther H. lips, Emma J. Groen, Marja C. J. van Oirsouw, Shelley E. Hwang, Joseph Y. Lo, Jelle Wesseling, Ritse M. Mann, Jonas Teuwen, Grand Challenge PRECISION Consortium Steering Group, Alastair Thompson, Serena Nik-Zainal, Elinor J. Sawyer, Helen Davies, Andrew Futreal, Nicholas Navin, E. Shelley Hwang, Jos Jonkers, Jacco van Rheenen, Fariba Behbod, Marjanka Schmidt, Lodewyk F.A. Wessels, Daniel Rea, Proteeti Bhattacharjee, Hilary Stobart, Hilary Stobart, Deborah Collyar, Donna Pinto, Ellen Verschuur, Marja van Oirsouw

**Affiliations:** 1 Division of Molecular Pathology, the Netherlands Cancer Institute, Amsterdam, Netherlands; 2 Department of Surgery, the Netherlands Cancer Institute – Antoni van Leeuwenhoek, Amsterdam, Netherlands; 3 Department of Health Services and Technology Research, Technical Medical Centre, University of Twente, Enschede, The Netherlands; 4 Borstkanker Vereniging Nederland, Utrecht, The Netherlands; 5 Department of Surgery, Duke University Medical Center, Durham, NC USA; 6 Department of Radiology, Duke University Medical Center, Durham, NC USA; 7 Department of Pathology, the Netherlands Cancer Institute – Antoni van Leeuwenhoek, Amsterdam, the Netherlands; 8 Department of Pathology, Leiden University Medical Center, Leiden, the Netherlands; 9 Department of Radiology, the Netherlands Cancer Institute – Antoni van Leeuwenhoek, Amsterdam, the Netherlands; 10 Department of Medical Imaging, Radboud University Medical Center, Nijmegen, the Netherlands; 11 Department of Radiation Oncology, the Netherlands Cancer Institute – Antoni van Leeuwenhoek, Amsterdam, the Netherlands; 12 Department of Radiology, Memorial Sloan Kettering Cancer Center, New York City, USA; 13 Netherlands Cancer Institute – Antoni van Leeuwenhoek Hospital, Plesmanlaan 121, Amsterdam, 1066 CX The Netherlands

**Keywords:** DCIS, DCIS grade, Invasive breast cancer, Active surveillance, Artificial intelligence, Deep learning

## Abstract

**Background:**

Ductal Carcinoma In Situ (DCIS) can progress to invasive breast cancer, but most DCIS lesions never will. Therefore, four clinical trials (COMET, LORIS, LORETTA, AND LORD) test whether active surveillance for women with low-risk Ductal carcinoma In Situ is safe (E. S. Hwang et al., BMJ Open, 9: e026797, 2019, A. Francis et al., Eur J Cancer. 51: 2296–2303, 2015, Chizuko Kanbayashi et al. The international collaboration of active surveillance trials for low-risk DCIS (LORIS, LORD, COMET, LORETTA),  L. E. Elshof et al., Eur J Cancer, 51, 1497–510, 2015). Low-risk is defined as grade I or II DCIS. Because DCIS grade is a major eligibility criteria in these trials, it would be very helpful to assess DCIS grade on mammography, informed by grade assessed on DCIS histopathology in pre-surgery biopsies, since surgery will not be performed on a significant number of patients participating in these trials.

**Objective:**

To assess the performance and clinical utility of a convolutional neural network (CNN) in discriminating high-risk (grade III) DCIS and/or Invasive Breast Cancer (IBC) from low-risk (grade I/II) DCIS based on mammographic features. We explored whether the CNN could be used as a decision support tool, from excluding high-risk patients for active surveillance.

**Methods:**

In this single centre retrospective study, 464 patients diagnosed with DCIS based on pre-surgery biopsy between 2000 and 2014 were included. The collection of mammography images was partitioned on a patient-level into two subsets, one for training containing 80% of cases (371 cases, 681 images) and 20% (93 cases, 173 images) for testing. A deep learning model based on the U-Net CNN was trained and validated on 681 two-dimensional mammograms. Classification performance was assessed with the Area Under the Curve (AUC) receiver operating characteristic and predictive values on the test set for predicting high risk DCIS-and high-risk DCIS and/ or IBC from low-risk DCIS.

**Results:**

When classifying DCIS as high-risk, the deep learning network achieved a Positive Predictive Value (PPV) of 0.40, Negative Predictive Value (NPV) of 0.91 and an AUC of 0.72 on the test dataset. For distinguishing high-risk and/or upstaged DCIS (occult invasive breast cancer) from low-risk DCIS a PPV of 0.80, a NPV of 0.84 and an AUC of 0.76 were achieved.

**Conclusion:**

For both scenarios (DCIS grade I/II vs. III, DCIS grade I/II vs. III and/or IBC) AUCs were high, 0.72 and 0.76, respectively, concluding that our convolutional neural network can discriminate low-grade from high-grade DCIS.

## Highlights

• Artificial intelligence could play a role in discriminating high- from low-risk DCIS.

• The developed CNN could fairly discriminate high- from low-risk DCIS and/or IBC.

• The NPV 0.84 may be clinically relevant for DCIS active surveillance trials.

## Key finding

• An AUC of 0.72 was achieved on the test-set, with a PPV 40.3%, a NPV of 90.9%. In the upstaged scenario, thus low-risk DCIS versus high-risk DCIS and/or IBC, the AUC increased to 0.76 in the test set, with a PPV of 80.0% and NPV value of 83.9%.

## Importance

• The CNN could be a supportive tool in combination with other clinicopathological factors, to personalize treatment in patients with DCIS.

## Introduction

At present, about 20% of all newly screen-detected ‘breast cancers’ are in fact Ductal Carcinoma In Situ (DCIS) [1, 2]. DCIS is an intraductal proliferation of neoplastic cells with the absence of invasion into surrounding stromal breast tissue. Nonetheless, some DCIS lesions advance to invasive breast cancer (IBC) when left untreated [3–6]. Only a minority (~ 10%) of the DCIS lesions cause clinical symptoms (i.e. palpable mass, or bloody nipple discharge). The majority of DCIS is therefore detected on screening mammography, by the identification of associated calcifications (~ 90%) [7–10].

Since DCIS is considered to be a potential precursor of IBC, treatment of DCIS should prevent women from progression of DCIS to IBC. As a result, over the last decades, women with DCIS have been treated by breast-conserving surgery, often followed by radiotherapy, or even mastectomy, in some countries regularly supplemented with endocrine treatment. As the incidence of advanced stages of IBC has not decreased, however, the current therapeutic approach for screen-detected DCIS consists, at least partly, overtreatment [11, 12].

Currently, four active surveillance trials (COMET, LORIS and LORD, LORETTA–trial) [13–16] are evaluating the safety of active surveillance for low-risk, defined as grade I or II DCIS and, for the LORD-trial, being estrogen receptor positive and HER2-negative as well. So, grade is an essential eligibility criterium for the active surveillance trial, as it is a strong predictor of prognosis [15, 17, 18], indicating the importance for appropriate differentiation in DCIS grade.

If these active surveillance trials indeed show that it is safe to leave low-risk DCIS in situ, it would be beneficial to determine the grade of DCIS based on mammography since histopathological diagnosis will be based on biopsy only in active surveillance patients. It may thus not properly document the heterogeneity within the lesion based on a limited tissue sample from the biopsy only, and may miss higher grade areas or invasive foci. However, radiologists have so far not been able to adequately predict occult invasive disease when DCIS presents as calcifications, let alone determine the grade of eventual DCIS from the appearance of calcifications [19, 20]. A recent study showed that radiologists were able to predict invasive disease when DCIS presents as calcifications better than chance, where accuracy increased particularly for smaller DCIS lesions (< 2 cm) and after exclusion of microinvasive disease [21]. However, this not consistent enough to rely on in daily clinical practice.

As there are clear indications that the shape and distribution of the calcifications are associated to the aggressiveness of the lesion [10, 22, 23] the inability of radiologists to distinguish between high- and low-risk DCIS may be due to the large inter-rater variability of both radiologists and pathologists [24–27]. For example, observers generally agree on the presence or absence of a mass or calcifications but disagree on calcification descriptors [24, 28–32]. More importantly, calcification descriptors are associated with DCIS grade. However, large reader variability excists in reporting calcification descriptors, making it challanging for radiologists to report DCIS grade based on these calcification descriptors [19, 20]. For instance, Roos et al. found that inear microcalcifications were significantly associated with high grade DCIS, while presence of fine granular calcifications was more often associated with lower grade [20].

Available Computer-Aided Detection (CADe) and Computer-Aided Diagnosis (CADx) algorithms were developed to support radiologists in assessment of mammograms. Several studies have been performed using CADe and CADx for calcification segmentation and detection on mammography [33–36], mainly to prevent overlook errors of radiologists. Other studies using CADe/CADx more specifically evaluated DCIS [37–39], focusing on segmentation of calcifications and prediction of occult invasive disease with DCIS [40].

Image analysis enhanced with artificial intelligence can be categorized into two approaches which transform imaging information into mineable data [41, 42]. Both approaches have the same underlying concept of identifying and encoding simple patterns and many higher-order patterns of imaging features that are not visible with the naked eye. These features can be extracted from biomedical images (i.e. mammography) and be linked with clinical variables of interest (i.e. patient characteristics, clinical outcomes, tumor grade and tumor stage), enabling improved decision support [41, 42]. Generally, CAD systems use handcrafted features coupled with imaging features extracted with machine learning, to identify a phenotypical fingerprint. On the contrary, a CNN, which is a deep learning method, based on a complex network, inspired by the human brain architecture, is able to learn high-level features automatically from obtained images such as mammographies [43, 44]. Predicting tumor grade by utilizing a CNN on biomedical images have been studied earlier. For example, a study evaluated the diagnostic performance of a CNN for bladder cancer grading. The CNN was able to predict tumor grade based on tumor color, and achieved an accuracy of 94.1% to distinguish between low-grade and high-grade tumor using white light images [45]. In addition, another study predicting grade using a CNN, achieved accuracy of 90% in classifying meningioma grades based on MRI images [46].

In pursuit of reducing overtreatment of DCIS patients, the current study aims to identify a series of systematic differences in imaging characteristics by utilizing a CNN, and to investigate whether the CNN was able to separate high-risk from low-risk DCIS, and to improve the discrimination of DCIS with and without invasive components. To bridge the gap between daily clinical practice and risk of overtreatment of DCIS, a CNN might be a substantial support to clinicians. Therefore, we explored whether the CNN could be used as a decision support tool in order to facilitate active surveillance in patients with biopsy proven DCIS eligible for active surveillance trials.

## Methods and materials

### Patient selection

**Fig. 1:**
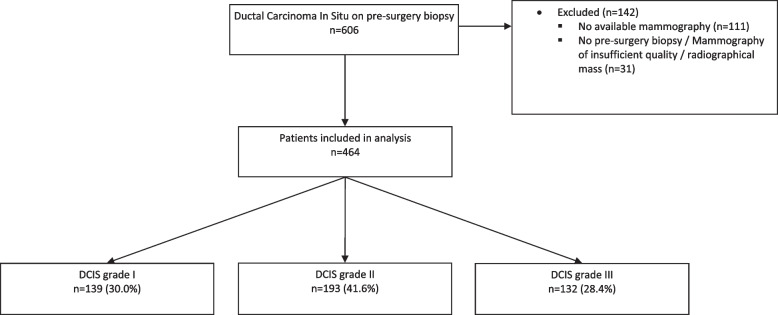
Flow-chart patient inclusion. DCIS, Ductal Carcinoma in Situ. Included patients were those diagnosed with DCIS between 2000 and 2014 whose initial biopsy was performed at the Netherlands Cancer Institute – Antoni van Leeuwenhoek Hospital or who were referred to the Netherlands Cancer Institute – Antoni van Leeuwenhoek Hospital for a second opinion

The current study population consists of women, aged 18 years or older, diagnosed with DCIS between 2000 and 2014 whose initial biopsy was performed at the Netherlands Cancer Institute – Antoni van Leeuwenhoek Hospital or who were referred to the Netherlands Cancer Institute – Antoni van Leeuwenhoek Hospital for a second opinion. Patients were eligible if pure DCIS was diagnosed on initial biopsy (vacuum 9G or core-needle biopsy 14G, the cases in this study have been collected over a long span of time and historical cases with 14G sampling have been included), and the pre-biopsy digital mammogram (Full-Field Digital Mammography, FFDM) was available, both screen and non-screen detected patients were included. Patients were excluded if lobular carcinoma in situ was reported, or when there was suspicion for, or evidence of, IBC on pre-surgery biopsy, or when there was a visible mass or architectural distortion on mammography. In this dataset all patients underwent definitive surgery. According to the Dutch guidelines patients with DCIS do not receive neoadjuvant hormone therapy or chemotherapy. Initially 606 DCIS patients were identified. A total of 142 patients were excluded for the following reasons: mammography not available (*n* = 111), insufficient quality of the obtained mammography and/or presence of radiological mass (*n* = 31). Ultrasound is routinely used to identify disease suspicious for invasion / solid high-risk DCIS, considering that patients with a mass were excluded, no data regarding ultrasounds was collected. After exclusion, 464 patients were eligible and included in this study, representing 854 unique images (392 Mediolateral-Oblique (MLO)and 386 CranioCaudal (CC) view, 76 other; i.e. true lateral views (ML and LM views), exaggerated craniocaudal views (XCCL), rolled lateral (RL), tangential (TAN) and lesion localization (LL) views). Magnification views were not included for analysis (Fig. 1).

### Data pre-processing and augmentation

Clinical information including patient age, localization, lesion size, and grade of primary DCIS lesion were extracted from the electronic patient record. Biopsies showing DCIS were selected through the Netherlands nationwide registry of histology and cytopathology records (PALGA) [47] and through the regional tumor registry at the Netherlands Cancer Institute—Antoni van Leeuwenhoek Hospital (NKI–AVL). These pre-surgical biopsies were either performed at NKI–AVL or were taken at another hospital and sent for routine second opinion to NKI–AVL. Information regarding localization, including approximate site, was used to manually annotate the calcified regions on the mammograms. In case of multiple groups of calcifications, the whole area was annotated. However, in case of multiple separate calcifications clusters, the cluster that was described in the radiology report as the biopsied area was annotated. In case of extensive calcifications the whole area was annotated. All calcified regions were manually annotated by two trained readers on the full image (SA: MD, MM: Technical Medicine researcher), supervised by a dedicated breast radiologist (RM) using 3D Slicer, version 4.10.2. Mammographies were split on a patient level and calcified regions were annotated once by one of the two trained readers, the two readers did consult each other if needed. Training and test sets were split randomly and stratified for grade. Study approval was granted by the IRB of our institute, 19.050/IRBd19016.

The histopathological report after surgical resection was considered the ground truth and accordingly patients were separated in pure low-risk DCIS (grade I/II) and grade III DCIS and/or invasive disease groups.

Before feeding images into the neural network, several augmentation operations were applied. Each image had one of the DICOM lookup tables provided by the vendor randomly applied and was subsequently linearly rescaled to the range [0, 1]. Subsequently, the images were cropped to a shape of 1024 × 1024 around the center of the lesion which was randomly perturbed by at most 150 pixels in both directions. Other data augmentations were a random horizontal flip (*p* = 50%), a random gamma transform with gamma parameter between 0.95 and 1.05, and finally, Gaussian additive noise was applied with a magnitude which was at most 5% of the pixel intensity value.

### Network architecture

**Fig. 2:**
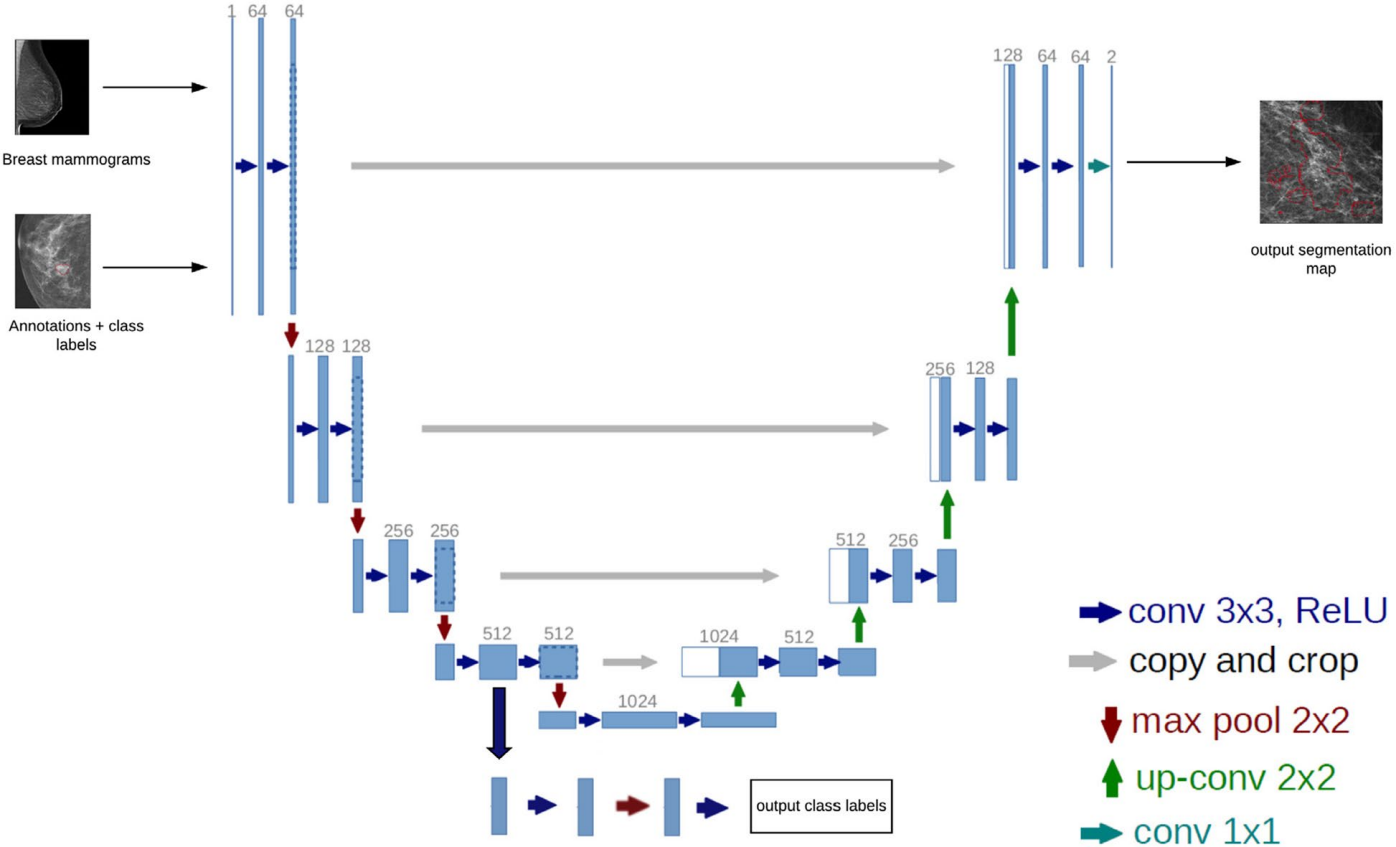
U-Net architecture with the segmentation and classification branches. Using mammography images as input, the segmentation branch is used to for segmentation of calcifications, whilst the classification branch is used to distinguish low-risk from high-risk DCIS

 A novel fully convolutional neural network (fCNN) was designed to combine both segmentation of the lesion and image-level classification. The proposed network is based on the well-known U-Net architecture, first described by Ronneberger [48], which is an encoder-decoder architecture. In addition, we attached an extra convolutional branch at the bottleneck to perform classification into grade I/II or grade III DCIS /invasive breast cancer. The U-Net part of the complete network is of depth five, where each block in the encoder path consists out of a block containing convolutions, followed by a rectified linear unit (ReLU) and a max pooling operation for downsampling. During upsampling in the decoder pathway of the network, the max pooling operations were replaced by bilinear interpolation operations. The downsampling and upsampling pathways shared information using skip-connections by combining the low-level yet high-resolution features from the encoder pathway with the location information of the decoder pathway to compute global information and provide the network with the ability to compute high-resolution segmentation masks. The final segmentation output is generated resulting from a set of three final convolution operations see Fig. 2.

The classification branch of the network is constructed to classify the original input image as low-risk or high-risk DCIS. Connected at the final layer of the encoder, the classification branch consists of a single convolution followed by a double convolutional block and a final output convolutional layer. The classification of the input image is provided by a softmax function [49].

The final loss was the sum (equally balanced) of the segmentation and classification loss. The total loss consisted of the sum of the focal loss function for the image classification, and a top-k cross entropy to compute the loss of the segmentation branch. The difference between a normal cross-entropy loss and the top-k cross entropy loss is that only for the top-k worst classified pixels the loss is computed and backpropagated.

To train the network we used the Adam optimizer with a starting learning rate of 0.00025, where each 150 iterations, the learning rate was decreased by a factor of 0.5. We have selected the batch size to maximize the GPU memory usage and have adapted the learning rate accordingly’ For our hardware this led to a batch size of 16.

**Fig. 3:**
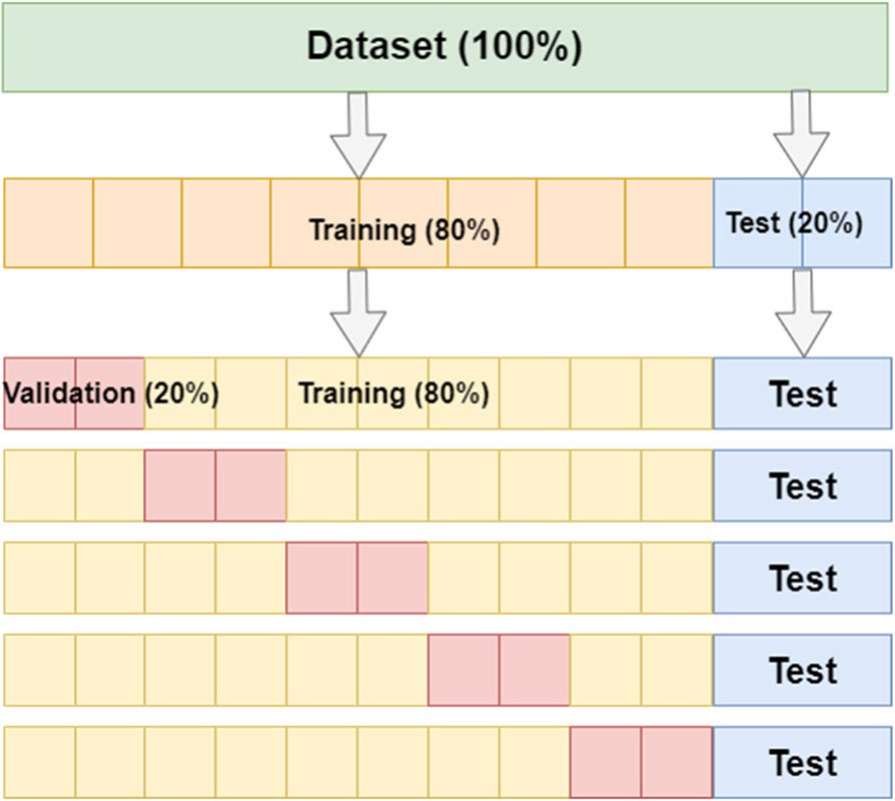
Overview of data partitioning

The collection of mammography images was partitioned on a patient-level into two subsets, one for training containing 80% of cases (371 cases, 681 images) and the remaining 20% (93 cases, 173 images) for testing. The training subset was further divided into five folds, each fold containing a random selection of 80% of the patients for training and the other 20% for validation, see Fig. 3. The final prediction was the average of the combined MLO and CC view output probabilities of the same patient.

### Statistical analysis

To evaluate the performance of the network in segmenting and classifying the image, we used the dice similarity coefficient (DICE) and the area under the receiver operator characteristic (AUROC) [50], respectively. Given an input image, the network produces a probability to which different discrimination thresholds were applied in order to predict class membership, reflected in the ROC curve. The clinical translation of the AUC values are as follows; a higher AUC value corresponds with a greater accuracy in predicting high-risk (grade III) DCIS. In the scenario where upstaging to invasive disease was evaluated by the CNN, a higher AUC value translated a greater accuracy in predicting high-risk (grade III) DCIS, and/or the presence of invasive disease. The final surgical specimen was the leading diagnosis as incorporated in the CNN model. In addition, positive predictive value (PPV) and negative predictive value (NPV) were calculated as secondary performance metrics to assess the performance of the classifier. Positive indicated DCIS that was upgraded to higher (III) grade DCIS and / or upstaged to invasive cancer at the time of surgery. Negative corresponded to pure DCIS without upstaging or upgrading to higher grade DCIS. It was assumed that from a likelihood of 0.5 for high-grade and/or invasive disease watchful waiting would be deemed too dangerous, hence NPV and PPV were calculated using this threshold. To this end we trained and tested the network to evaluate whether it was able to classify DCIS grade, in order to explore whether it is feasible that the network could be applied as an support decision tool next to classical histopathological assessment of DCIS grade. Training and testing of the network was performed on a NVIDIA Tesla T4 GPU (Nvidia Corporation, Santa Clara, California, United States). The deep learning network was implemented using Pytorch 1.5 and Python 3.7.

## Results

**Fig. 4:**
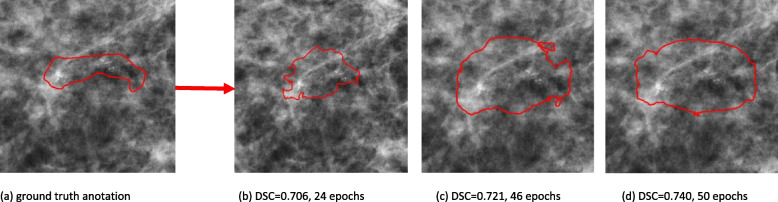
Improvement of calcification segmentation for different eppochs: Demonstratinghow the segmentation performance by the network improved during training, Fig. 4a gives an example of a ground-truth annotation that was used to train the network. A clear improvement in segmentation performance can be seen in Fig. 4b-d, where in the beginning stages of training (Fig. 4b), the network did not include all calcifications. However, a more extensive and smoothly coverage of the calcifications can been seen as the data is further processed, Fig. 4c and d. **a** ground truth anotation. **b** DSC=0.706, 24 epochs. **c** DSC=0.721, 46 epochs. **d** DSC=0.740, 50 epochs

The median age of the included patients was 54 years (interquartile range 49–62) and approximately half (51.9%) of the patients were post-menopausal at diagnosis. Furthermore, over half (53.9%) of the DCIS lesions were detected during population screening. Mean lesion size was 29.8 mm, and approximately 70% of the patients had a low-risk DCIS lesion (grade I/II). Among those for whom information was available 225 (48.5%) patients were diagnosed using a 9G vacuum-assisted biopsy and 92 (19.8%) using a 14G core-needle biopsy, for 147 (31.7%) patients the method of biopsy that was used was unknown. A total of 47 (14.2%) patients were upgraded to an higher grade DCIS based on the final surgical excision specimen. Seventeen (36.2%) patients with grade I DCIS on biopsy were grade II DCIS on surgical resection specimen, two (4.3%) patients with grade I DCIS on biopsy had grade III DCIS on resection specimen and 28 (59.5%) patients had initially grade II DCIS on biopsy but grade III DCIS on final excised specimen. When sub classifying according to low and high-risk DCIS, 30 (63.8%) patients who had a primary diagnosis of low-risk (grade I/II) DCIS, were upgraded to high-risk DCIS upon examination of final surgical excision specimen. Additionally, 68 (14.7%) patients were upstaged, meaning that these patients harbored occult invasive disease, initially not diagnosed on pre-surgical biopsy, but determined on final surgical specimen. Of these 68 upstaged patients, 46 (67.6%) patients were initially diagnosed as low-risk (grade I/II) DCIS, whereas 22 (32.4%) patients who were upstaged, had an initial diagnosis of high-risk (grade III) DCIS. Table 1 shows patient and tumor characteristics (Fig. 4).

**Table 1:** Patient and tumor characteristics (*n* = 464)

Age at diagnosis	n (%)
20–49	146 (31.5)
50–59	186 (40.1)
50–69	96 (20.7
70+	36 (7.8)
Median (interquartile range)	54 (49–62)
**Menopausal status**	
Pre-menopausal	121 (26.1)
Peri-menopausal	40 (8.6)
Post-menopausal	241 (51.9)
Unknown	62 (13.4)
**Method of detection**	
Screen-detected	250 (53.9)
Symptomatic	53 (11.4)
Unknown^a^	161 (34.7)
**Lesion size (mammography, mm)**	
0–19	158 (34.1)
20–50	109 (23.5)
≥ 50	79 (17.0)
unknown	118 (25.4)
Mean (± standard deviation)	29.8 (25.2)
**Grade (based on surgical specimen)**	
I	139 (30.0)
II	193 (41.6)
III	132 (28.4)
**Upstage to IBC**	
Yes	68 (14.7)
No	396 (85.3)

^a^Including 110 diagnosed during follow-up for a previously treated breast lesion and 51 referred for routine second opinion

**Fig. 5:**
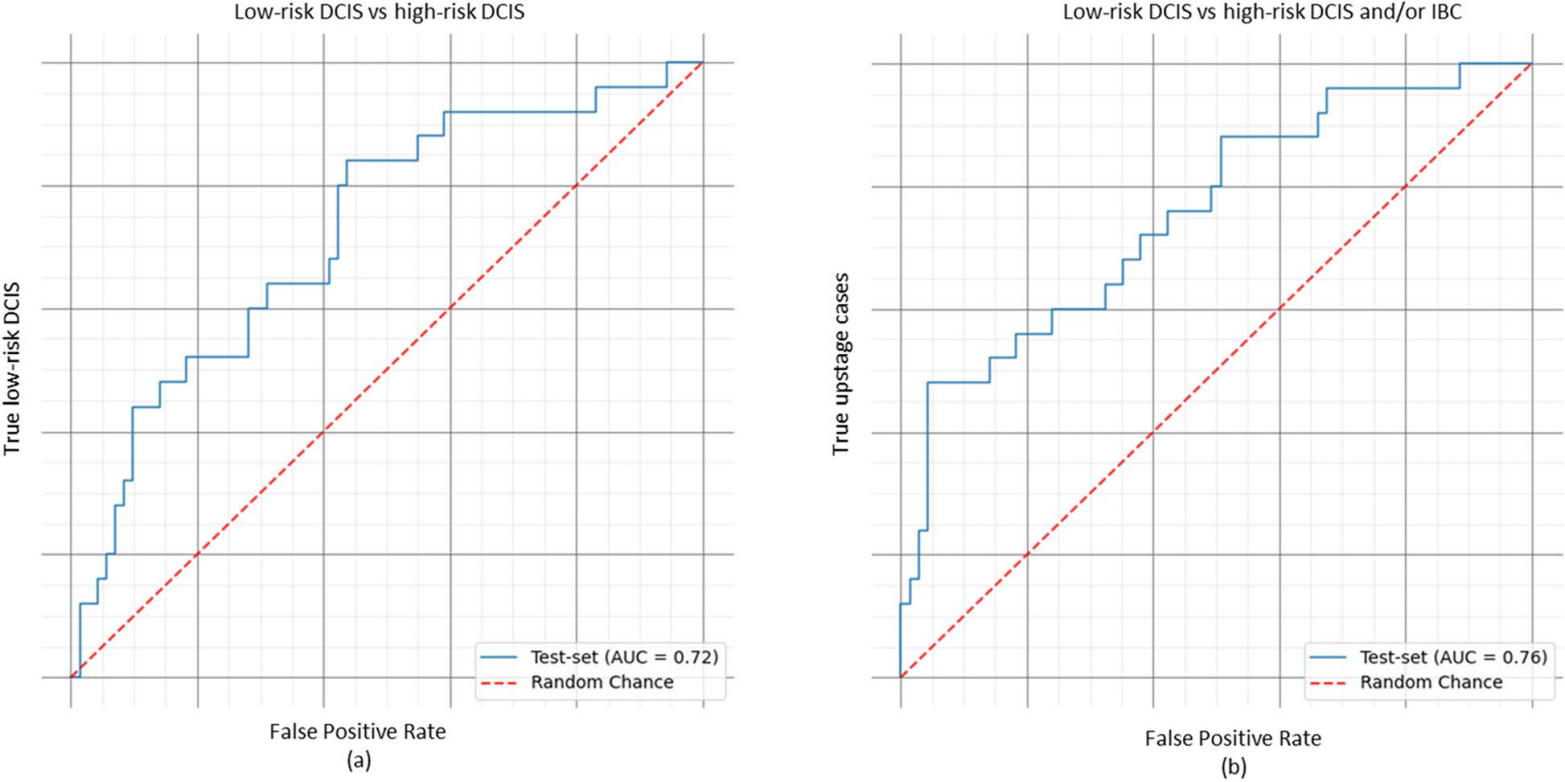
Test-set, ROC curve AUC. **a** Classification of low-risk (grade I/II) DCIS vs high-risk (grade III) DCIS. **b** Classification of low-risk (grade I/II) DCIS vs high-risk (grade III) DCIS and/or IBC

The 464 included patients yielded 854 mammograms which were used for our fCNN algorithm. The network was trained using five folds for 500 epochs each. In the pure DCIS cases (thus excluding the upstaged cancers) where we aimed to discriminate low-risk DCIS from high risk, there were 93 cases in the test set. Overall, an AUC of 0.72 was achieved on the test-set, corresponding with a positive predictive value 40.3%, a negative predictive value of 90.9%. In the upstaged scenario, thus low-risk DCIS versus high-risk DCIS and/or IBC, the AUC increased to 0.76 in the test set. In this scenario a positive predictive value of 80.0% and a negative predictive value of 83.9% were determined. See Fig. 5 for ROC curve and AUC classification.

## Discussion

This study demonstrated that it is feasible to discriminate high- from low-risk DCIS, by applying a deep learning network on pre-surgical mammographies showing only calcifications. After external validation, the network could be applied as an extra decision support tool in patients opting for participation in the active surveillance trials. Thus, offering further refining of clinical decision making and treatment planning, together with the classical histopathological assessment of DCIS grade.

For network optimization, the network was first trained as a stand-alone U-Net architecture for the image segmentation task only. During optimization, the network parameters were tuned to maximize dice scores estimated on the validation set. With the best performing parameters, the classification branch was added to train the network for the classification task. This classification task achieved an AUC of 0.72 on the test-set, which excluded the upstaged cases. Positive predictive value was 40.3% and negative predictive value was 90.9%. In the clinically more realistic scenario where the upstaged cases were included the AUC was even higher, at 0.76, with a positive predictive value of 80.0% and a negative predictive value of 83.9% based on the used cut-off. Our results show that the network could discriminate high-grade from low-grade DCIS. More importantly, the demonstrated NPVs are clinically relevant considering the relative low risk of including a high-risk DCIS patient for active surveillance.

Previous studies investigating mammographic image data to predict the presence of occult invasive breast disease next to the presence of DCIS demonstrated good performances [39, 40, 51, 52]. In one study where deep features were extracted from digital mammograms using deep convolutional network, pre-trained on non-medical images, to predict the presence of occult invasive disease in patient with DCIS, an AUC of 0.70 (95% CI, 0.68–0.73) was achieved, which is comparable with the current study [51]. However, the main aim of the current study was to develop a CNN that is also able to differentiate low-risk (grade I/II) DCIS from high-risk (grade III) DCIS and invasive disease. The demonstrated negative predictive values of 90.9 and 83.9% are promising in guiding patients who are opting for active surveillance. The network could be applied as an extra safety measure before inclusion in active surveillance trials, where it can be utilized for definitive grading of DCIS. The most likely clinical scenario for our classifier is the scenario where upstaging is possible, as we would apply it on pre-surgical mammograms, where occult invasive disease could be present.

This study has several limitations and strengths. First, the main aim of this study has been the successful classification of calcifications on mammograms. Major inclusion criteria for the active surveillance trials is that the DCIS is detected by screening, in this cohort screen-detected status could be confirmed in only 54%. However, an additional 51 patients were referred to our hospital for a routine second opinion and while the majority of these patients likely also had screen-detected DCIS, we cannot exclude other means of detection. Thereby, the distribution of DCIS grade in the current study is different compared to an earlier study [53] performed by our research group. Meaning that the current study is less reflective of a true screen-detected cohort of DCIS patients. Another limitation is that the manual calcification labeling was not performed by radiologists, but by trained researchers. Although intensively supervised by a dedicated breast radiologist, this might have affected the quality of labeling. Nevertheless, as the segmentation is merely meant as a proxy task for the classification, the impact of an non-perfect segmentation model is minor. In practice, a significant inter-rater variability is also seen when radiologists perform this task [24]. Also, we did not perform a risk-benefit analysis to demonstrate the performance of the CNN compared to traditional biopsies for DCIS grade assessment or upstage rate. However, to facilitate active surveillance for DCIS patients, we explored the feasibility of the CNN as an extra safety measurement in addition to the classical histopathological assessment of DCIS grade and upstage rate. Therefore we believe that the clinical application of our CNN could only be applied after external validation and ideally with a risk-benefit analysis comparing the accuracy of the CNN with biopsies. A strength of this study is, that to our knowledge this is one of the largest datasets available to address the specific research question of identifying and classifying the grade of DCIS based on image features alone. However, this remains a relatively small data set with almost half the cases entered twice (CC and MLO). Furthermore, our CNN incorporated the segmentation and the classification in one neural network. Thereby we demonstrated high AUC of 0.72 for DCIS low-risk versus high-risk, and even a higher AUC of 0.76 for low-risk versus high risk DCIS and/or IBC.

## Conclusion

In conclusion our AUC for both models were high and we conclude that our CNN is a good discriminator of high- and low-grade DCIS. Furthermore, by adding the occult IBC to the CNN, we achieved even a higher AUC of 0.76, which is clinically relevant considering the shift in treatment strategy for low-risk DCIS. Following confirmation of the CNN in another independent dataset it could be a supportive tool in combination with other clinicopathological factors to offer personalized treatment in patients with DCIS.

## Data Availability

The datasets used and/or analyzed during the current study are available from the corresponding author on reasonable request.
